# A Performance Evaluation Matrix for Measuring the Life Satisfaction of Older Adults Using eHealth Wearables

**DOI:** 10.3390/healthcare10040605

**Published:** 2022-03-23

**Authors:** Mei-Yuan Jeng, Tsu-Ming Yeh, Fan-Yun Pai

**Affiliations:** 1 Department of Life Sciences, National Open University, New Taipei City 247, Taiwan; 980020@gapps.nou.edu.tw; 2 Department of Industrial Engineering and Management, National Quemoy University, Kinmen 892, Taiwan; 3 Department of Business Administration, National Changhua University of Education, Changhua 500, Taiwan

**Keywords:** eHealth wearables, life satisfaction, older adults, performance evaluation matrix

## Abstract

eHealth wearables can track users’ state of health, record their physiological data, and facilitate self-care. In this study, we examined whether they enhance older adults’ casual exercise willingness and life satisfaction. After reviewing the related literature, the performance and satisfaction of elements for older adults to use eHealth Wearables were determined. The elements were derived from the means–end chain analysis. Three dimensions, product attributes, beneficial consequences, personal values, and responding elements, were identified first. The Performance Evaluation Matrix (PEM) was then established to determine the elements to be improved. A total of 250 questionnaires were distributed, out of which 206 valid questionnaires were completed and returned. In the proposed PEM, the product attributes that were in the priority improvement zone were accessibility, learnability, usability, affordability, positioning, pedometer, heart rate monitor, and data feedback. These elements are the most essential properties in need of improvement.

## 1. Introduction

Older adults have become the fastest-growing group in the world, a growth closely linked to advancements in technology and medicine. In response, governments worldwide are actively investing resources and formulating policies to meet the challenges of population aging [1]. According to statistics released by the World Health Organization (WHO), the global population is aging at an alarming rate, with the proportion of older adults over the age of 60 increasing from 8% in 1950 to 11% in 2011, and projected to reach 22% by 2050. Within the elderly population, the over-80 subgroup exhibits the fastest growth. It is expected that the global elderly population will surpass that of children (under 15) for the first time in 2045 [2]. The biggest social impact of such aging is the increased demand for medical assistance and long-term care. However, with the decline in birthrates in recent years, attempting to meet rising demands by allocating more labor and resources may not be a viable solution. With the rapid adoption of smart technologies in people’s daily lives, utilizing smart technologies, such as wearables, service robots, Internet of Things (IoT) applications, and other home devices to satisfy the demand and improve the quality of life of older adults, thereby alleviating the workload of caregivers while promoting the development of relevant industries, has become a crucial topic of discussion.

As population aging accelerates, the impact of older adult health on society will become increasingly manifest, and the promotion of older adult health, active aging, and disease prevention will assume greater importance. The purpose of promoting active aging is to improve people’s quality of later life and help them lead a safe, healthy, and social lifestyle [3]. Aging should not be a negative process; on the contrary, it is to be viewed as a positive life progression. Life satisfaction is widely adopted as a standard to evaluate many aspects of older adults’ lives, including health, psychology, and social engagement. It is an integral part of active aging and a key indicator of older adults’ quality of life [4]. Health is a prerequisite of a high level of life satisfaction, and regular physical exercise is essential for healthy aging. Exercise has been shown to improve physical, functional, mental, and cognitive health. For example, Yen and Chen [5] found that casually exercising for 150 min a week has positive health implications for older adults. Although mobility decreases with age, it is possible to improve that of older adults and encourage them to remain active through the power of technology.

eHealth wearables can track users’ state of health, including sleep conditions, calories burned, heart rate, and brain activity. In this study, we examine whether such wearables can improve the health and life satisfaction of older adults and serve as a useful tool in preventative medicine. Specifically, our objective is to determine whether such wearables (i.e., smartwatches and bracelets) enhance older adults’ willingness to engage in casual exercise (regular and speed walking), increase their life satisfaction, improve physical health, induce self-management of chronic diseases, and reduce medical resource utilization.

## 2. Literature Review

### 2.1. eHealth Wearables and Elders’ Health

eHealth wearables are small electronic health management devices that can be linked to smartphones or tablet computers. They help reduce medical resource costs and promote healthy lifestyles. The commercial potential of mobile medicine and eHealth wearables have skyrocketed in recent years with the sharp rise in the demand for chronic disease monitoring, long-term care, and self-health management [6,7]. Smart wearables are equipped with sensors and transmitters to monitor, collect, display, and transmit data automatically and perpetually [8]. They can also be worn for long periods without disrupting the user’s daily routine. There are many types of wearables, including glasses, watches, apparel, and other accessories, of which smartwatches and bracelets are currently the most commonly used. The functions of smart bracelets and smartwatches are similar: they primarily monitor and collect health data, such as tracking calories burned, steps taken, and sleep conditions. In view of the clear value of smartwatches and bracelets and their wide acceptance by the public, we selected smart bracelets as the target of research.

As an accessory of smartphones, eHealth wearables have immense market potential. The number of smartphones users in the world reached 2.1 billion in 2016. It was projected in the Statistical Number of Smartphone Users Worldwide from 2014 to 2019 to reach 2.5 billion by 2020. Bruijink et al. [9] reported that the number of downloads for health and fitness apps reached 165 million in 2015. These findings confirm the feasibility of using mobile health devices to monitor personal health and encourage self-health management.

A number of previous studies published the benefits of mobile health apps in managing a healthy lifestyle, such as managing diabetes [10], depression [11], and hypertension [12]. Burke et al. [13] found that using a personal digital assistant for diet management effectively reduced caloric intake. Schoeppe et al. [14] conducted a systematic review to examine the efficacy of interventions that use apps to improve diet, physical activity, and sedentary behavior and reported positive results. King et al. [15] also concluded that tablet-delivered social apps facilitate balance and strength training and that smartphone-delivered social apps support physical activity through social interaction. eHealth wearables provide users with a mobile physiological tracker to monitor their heart rate, heart rhythm, blood pressure, breathing, body temperature, and blood oxygen concentration [7,16]. They can be worn for long periods and are suitable for 24 h disease monitoring [6,17].

### 2.2. Life Satisfaction

The health of older adults is a major social concern since health is a prerequisite for leading a satisfactory later life. Exercise is recognized as a contributor to health, with many studies confirming that regular exercise reduces the risk of death, hypertension, coronary heart disease, Type II diabetes, obesity, colon cancer, breast cancer, osteoporosis, and depression. Conversely, the lack of physical activity increases the risk of chronic illness [18]. Thus, increasing the physical activity of older adults has become a major global public health objective [2]. Walking remains the most common form of casual exercise for such adults, and previous studies have shown that mild hikes have anti-aging effects and are a favorable form of casual exercise for less active older adults. Jeng et al. [19] examined a large number of older adults and found that regular exercise greatly improved cerebral blood flow, cerebral vasodilation, and brain health.

Life satisfaction refers to profound inner happiness in life based on a person’s experience of the external world. In other words, it represents a positive attitude towards life and reflects one’s feelings about the past, present, and future. Older adults with a high life satisfaction typically lead a healthy lifestyle [20,21], while those in poor health typically exhibit low life satisfaction. Thus, high life satisfaction becomes increasingly difficult to achieve as people grow older and develop health issues [22]. Sato et al. [23] examined 742 individuals and found that running improved life satisfaction.

## 3. Methodology

### 3.1. PEM Analysis

Lambert and Sharma [24] developed a Performance Evaluation Matrix (PEM) that characterized the importance of quality elements associated with logistics services and the performance of the enterprise with regard to these elements (i.e., customer satisfaction). PEMs are similar to importance–performance matrices in that both are two-axis matrices, with performance plotted along the X-axis and importance plotted along the Y-axis. However, PEMs differ in their approach by dividing importance/performance scores into three equal parts to generate more detailed results [25]. Based on these scores, PEMs can be divided into nine zones. The strategic significance and improvement priority of service elements and the strategies needed to improve them may differ depending on their position within the matrix, with the matrix, as a whole, illustrating the importance–performance relationships of various services [26].

**Figure 1:**
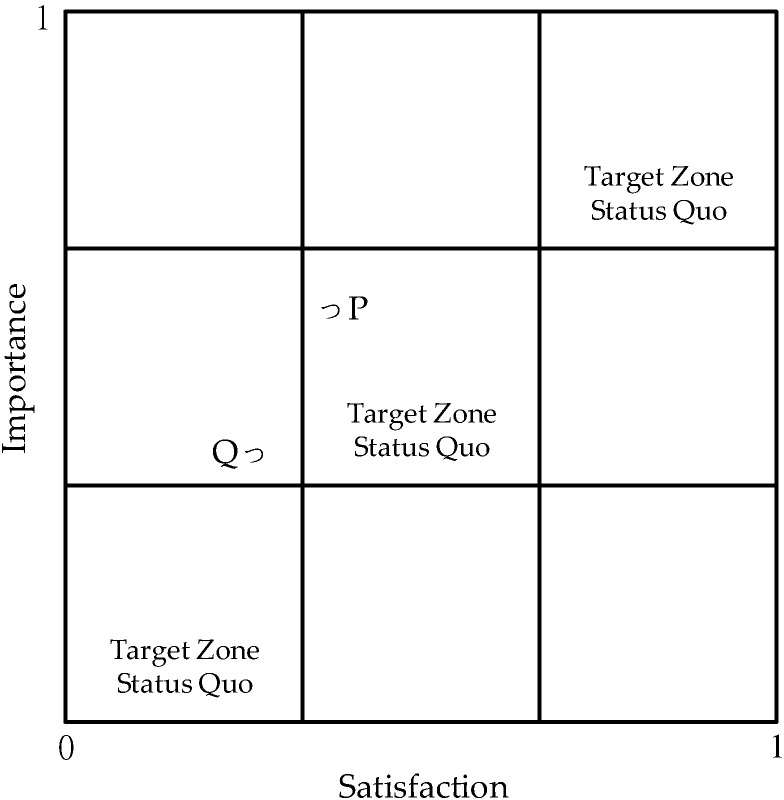
Performance Evaluation Matrix (PEM) [28]. P: status quo zone; Q: improvement zone.

Based on the PEM proposed by Lambert and Sharma [24], it is difficult to objectively determine whether to improve or promote the service items that fall within or close to the “moderate performance zones” (e.g., Coordinates P and Q in Figure 1; [27]). According to Lambert and Sharma [6], Coordinate P, which is in the “status quo zone,” and Coordinate Q, which is in the “improvement zone” (Figure 1), are the main improvement items. However, the positions of Coordinates P and Q in the matrix show that the importance of Coordinate P is significantly higher than satisfaction, suggesting that P should be an improvement item, while the importance and satisfaction of Coordinate Q are similar, suggesting that Q should maintain its status quo [26].

**Figure 2:**
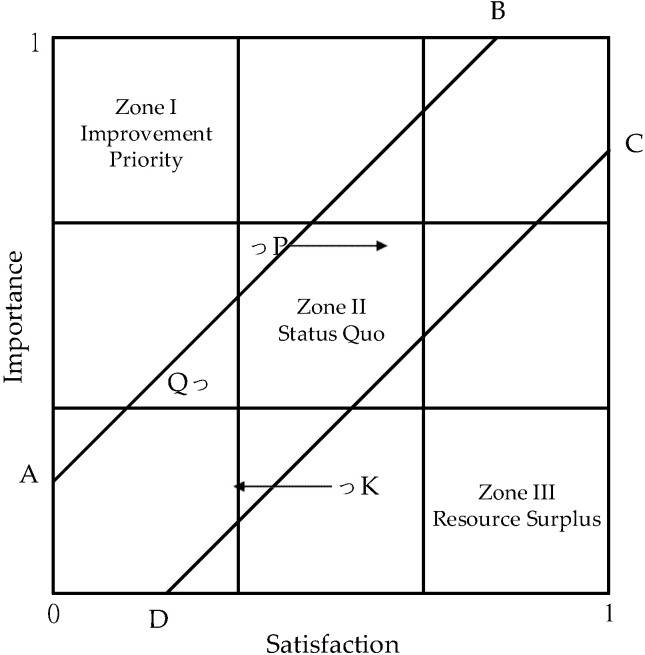
Controlled Performance Evaluation Matrix (PEM) [25].

In this study, we drew on the conclusions of Chen et al. [25], Yeh and Lai [27] and Hung et al. [28] to establish a set of controls to identify clearly the service items in need of improvement. In Figure 2, the coordinates of A, B, C, and D are A(0,d), B(1−d,1), C(d,0), and D(1,1−d), respectively. The A–B line and C–D line serve as the upper and lower control lines, demarcating Zone I, Zone II, and Zone III. When moderate performance principles are applied, Zone I would become the priority improvement zone, Zone II the status quo zone, and Zone III the resource surplus zone. In Figure 2, the position of P is in the priority improvement zone (Zone I), while that of Q is in the status quo zone (Zone II). Because these outcomes are different from those of Figure 1, we adopted the determination method of Figure 2 to ascertain the need to improve service items. In Figure 2, P and K are in Zone I and Zone III, respectively. Improving these items would cause them to shift vertically into Zone II. By establishing control lines, the ambiguity within the PEM can be eliminated [25].

**Figure 3:**
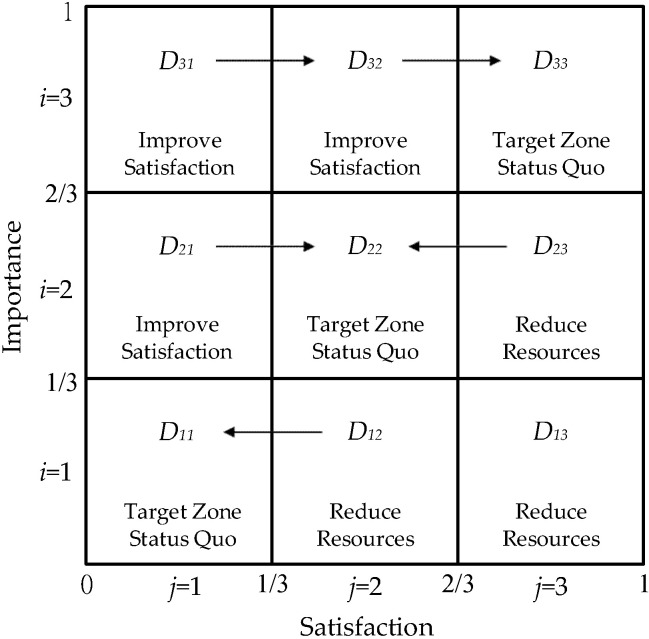
Performance Evaluation Matrix (PEM) [26].

In the PEM, X represented perceived importance for an element while Y represented satisfaction for the element. In using a K-scale to evaluate older adults’ perceived importance and satisfaction regarding eHealth wearables, the percentage index value for importance (*P_x_*) and that for satisfaction (*P_y_*) can be expressed as Equations (1) and (2).

(1) $${\;P}_{X}=\frac{\mu_{x}-min}{R}\;$$

(2) $$P_{y}=\frac{\mu_{y}-min}{R}$$

where $\mu_{y}$ and $\mu_{x}$ represent the mean value of satisfaction and importance, min = 1 represents the minimum value of the K-scale, and R = K−1 represents the range of the K-scale. Based on the above equations, the two percentage index values (*P_x_* and *P_y_*) were within an interval of [0, 1]. Therefore, the full range of a quintuple scale (K = 5) was R = K−1 = 4. When the perceived product/service importance or service satisfaction was higher than 3 (neutral), the index value was greater than 0.5. Conversely, when the perceived product/service importance or service satisfaction was lower than 3, the index value was less than 0.5. Thus, the index values clearly highlight the similarities or differences in the perceived product/service importance and satisfaction of the older adults. We then plotted the indices on the X-axis (satisfaction) and Y-axis (importance) of the PEM. The scope of the indices was within an interval of [0, 1]. We categorized the index values into low indices (0, 1/3), normal indices (1/3, 2/3), and high indices (2/3, 1) to form a matrix with nine performance zones, each representing a different level of performance, as illustrated in Figure 3. The zone symbols were expressed as Dip (i, j = 1,2,3), whereby D_11_, D_22_, and D_33_ were “target zones” defined as “moderate performance zones,” within which satisfaction and importance were deemed consistent. D_21_, D_31_, and D_32_ were the “high importance zones,” the items within which had high importance and low satisfaction and were the targets of improvement. D_12_, D_13_, and D_23_ were the high satisfaction zones, wherein items receive a surplus of resources and require resource redistribution to prevent wastage. Items within the target zones D_11_, D_22_, and D_33_ are recommended to maintain the status quo; those in D_21_, D_31_, and D_32_ lack resources and must be improved; those in D_12_, D_13_, and D_23_ receive a surplus of resources and better resource allocation is thus needed. Items falling outside the target zones can be improved by shifting them vertically into the target zones, as illustrated in Figure 3.

We redrew the performance control lines when incorporating the values into the PEM. First, we calculated the population mean and error values, which were expressed as $\mu_{\rho }$ and $\sigma_{\rho }$, respectively.

(3) $$\mu_{\rho }=\frac{\sum_{i=1}^{n}\left(R_{i}\right)}{n}\;$$

(4) $$\sigma_{\rho }=\sqrt{\frac{\sum_{i=1}^{n}\left(y_{i}{-x}_{i}\right)}{n}{-\mu }_{\rho }{}^{2}}=\sqrt{\frac{\sum_{i=1}^{n}{\left(R_{i}\right)}^{2}}{n}{-\mu }_{\rho }{}^{2}}$$

The upper and lower control lines previously defined were expressed as Equations (5) and (6).

(5) $$\mathrm{Upper}\;\mathrm{control}\;\mathrm{line}\;\mathrm{UCL}=\sqrt{\frac{\sum_{i=1}^{n}{\left(R_{i}\right)}^{2}}{n}{-\mu }_{\rho }{}^{2}}\;$$

Target T = 0

(6) $$\mathrm{Lower}\;\mathrm{control}\;\mathrm{line}\;\mathrm{LCL}=-\sqrt{\frac{\sum_{i=1}^{n}{\left(R_{i}\right)}^{2}}{n}{-\mu }_{\rho }{}^{2}}\;$$

We calculated $\mu_{\rho }$ and $\sigma_{\rho }$ using Equations (3) and (4). We then incorporated $\mu_{\rho }$ and $\sigma_{\rho }$ into Equations (5) and (6) to calculate the upper control line (UCL) and lower control line (LCL). Then, we calculated the $\mu_{\rho }$ and $\sigma_{\rho }$ of the importance–satisfaction items in the performance matrix using the above equations to determine the UCL and LCL. Once the control lines were included in the matrix, the $P_{x}$ and $P_{y}$ of the importance–satisfaction items were plotted in the PEM. In using the matrix, managers need only formulate improvement strategies to increase or reduce resources for the items outside the control lines to shift the items into the target zones.

### 3.2. Research Design

In this study, we adopted a quantitative questionnaire survey for data collection. We utilized the means–end chain method proposed by Jeng [19] to design the questionnaire and investigate the value of mobile health devices for older adults. We also grouped and reviewed the existing literature to formulate the questionnaire items. We divided the older adults’ perceptions of eHealth wearables into three variables, namely, product attributes, beneficial consequences, and personal values. The working definitions of the three dimensions are as follows:

(1) Product attributes: tangible or intangible elements such as product packaging, price, quality, brand, function, after-sales services, and vendor reputation;
(2) Beneficial consequences: users’ positive opinions of the product or service;
(3) Personal values: personal beliefs and desires to achieve specific life goals (psychological factors that motivate consumers to achieve important life goals).

Each dimension includes several elements. The dimension and responding elements are demonstrated in Table 1.

The questionnaire was divided into two sections: socioeconomic background and smart bracelet importance/satisfaction. The first section covered the respondents’ gender, age, family status, level of education, occupation, and disposable monthly income. The second section contained 32 items relating to the product attributes, beneficial consequences, and personal values of eHealth wearables (smartwatches and bracelets), as listed in Table 1.

We adopted a five-point Likert scale as the scoring system, with five options for indicating importance (“very important (5 points),” “important (4 points),” “neutral (3 points),” “unimportant (2 points),” and “very unimportant (1 point)”) and five options for indicating significance (“very satisfied (5 points),” “satisfied (4 points),” “neutral (3 points),” “unsatisfied (2 points),” and “very unsatisfied (1 point)”).

After the preliminary design of the questionnaire, we sent the questionnaire and procedure to the Research Ethics Committee of National Cheng Kung University, Taiwan, for ethical review. This study was conducted under approval number No. 108–184.

The subjects of this study were older adults over the age of 60 who are members of the Taichung Senior Citizen Active Learning Center and Taichung Evergreen Academy. We selected from the Center primarily members over the age of 60 and from the Academy those over the age of 65. Since all of the subjects were older adults, we visited the education venues in person to obtain the consent of the caretakers and subjects to participate in the study and to inform them about the research objectives and processes. We administered 250 questionnaires, out of which 44 invalid submissions were discarded, leaving 206 valid submissions, thus showing a recovery rate of 82%.

After the data were collected, the data were analyzed by PEM method to establish PEM.

## 4. Results

### 4.1. Background Variables

A total of 206 valid questionnaires were recovered. The demographics variables included gender, age, level of education, family status, occupation, and disposable monthly income. The respondents comprised 122 females (59%) and 84 males (41%), most of whom were between the ages of 66 and 70 (*n* = 71; 35%), followed by those between 71 and 75 (*n* = 69; 34%). The majority of the respondents were retirees (*n* = 143; 69%), followed by workers in the service industry (*n* = 21; 10%). In terms of educational level, most of the respondents were high school graduates (*n* = 78; 38%), followed by middle school graduates (*n* = 66; 32%). Other demographics-based rankings according to the sizes of the first two largest groupings included respondents who lived with family (*n* = 111; 54%) and those who lived with their spouses (*n* = 72; 35%); and respondents earning between TWD 40,001 and TWD 60,000 (*n* = 95; 46%) and those who earned between TWD 20,001 and TWD 40,000 (*n* = 92; 45%).

### 4.2. Reliability Analysis

Cuieford [32] attributed a Cronbach’s α coefficient of 0.7 or higher to high reliability, that between 0.35 and 0.7 to moderate reliability, and that of 0.35 or lower to low reliability, while Nunnally [33] asserts that variables with a Cronbach’s α coefficient of 0.7 or higher have acceptable reliability. In this study, the Cronbach’s α coefficients for product attributes (satisfaction), beneficial consequences (satisfaction), personal values (satisfaction), product attributes (importance), beneficial consequences (importance), and personal values (importance) were 0.631, 0.724, 0.747, 0.689, 0.688, and 0.822, respectively. The highest variable reliability coefficient was 0.822, and the lowest was 0.631, suggesting that the questionnaire was moderate to highly reliable (Table 2).

### 4.3. Overall PEM Analysis

Following the questionnaire survey, data were calculated with Equations (1) and (2) in Table 3. Then the control lines were defined (Table 4) to illustrate in Figure 3, which was used to identify the items in need of improvement.

First, Equations (3) and (4) were used to calculate the population mean and error values of the 32 importance–satisfaction items. In the matrix composed of importance and satisfaction indicators, the population means and error values were 4.010 and 0.136, respectively, which were then incorporated into Equations (5) and (6) to calculate the standard deviation of the UCL (+0.136) and LCL (−0.136) one time, as shown in Table 4.

**Figure 4:**
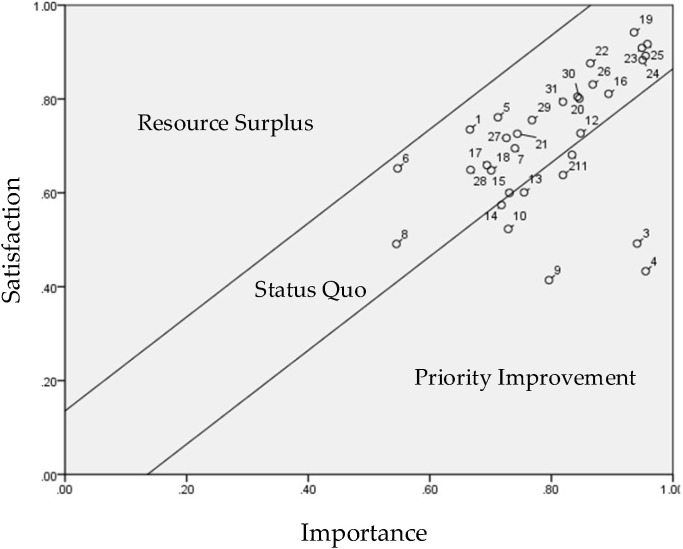
Overall Performance Evaluation Matrix (PEM).

The values in Table 4 were incorporated into the PEM (Figure 4) to identify the items in need of improvement. The results indicated eight items in the priority improvement zone: accessibility (2), learnability (3), usability (4), affordability (9), positioning (10), pedometer (11), heart rate monitor (13), and data feedback (14).

The results also showed that the eight abnormal nodes outside the control lines were all related to product attributes.

In addition to creating a PEM to highlight older adults’ use of eHealth wearables, we also created PEMs for the gender (male and female) and age (younger and older than 70) demographic variables to determine whether different groups of older adults had different preferences concerning the use of eHealth wearables.

### 4.4. PEM Analysis for Gender

**Figure 5:**
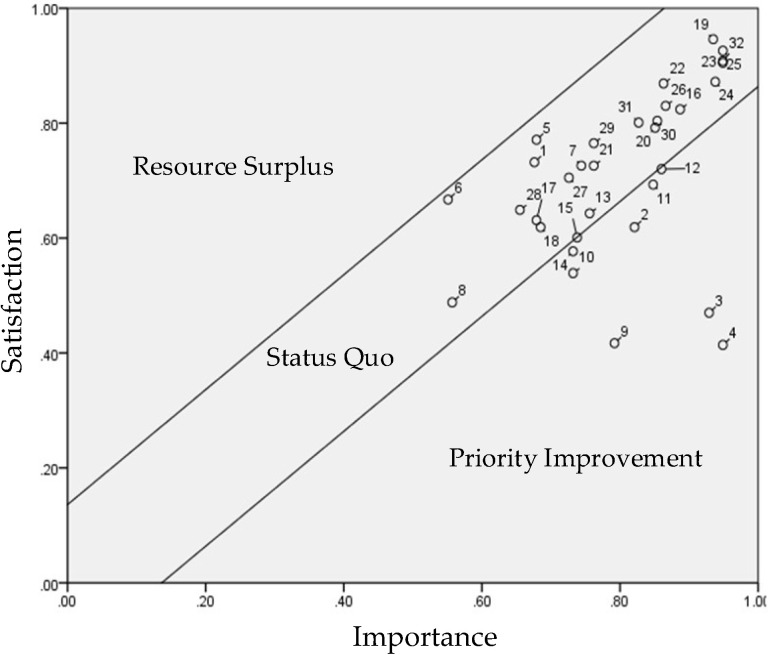
Performance Evaluation Matrix (PEM) of the Male Respondents.

**Figure 6:**
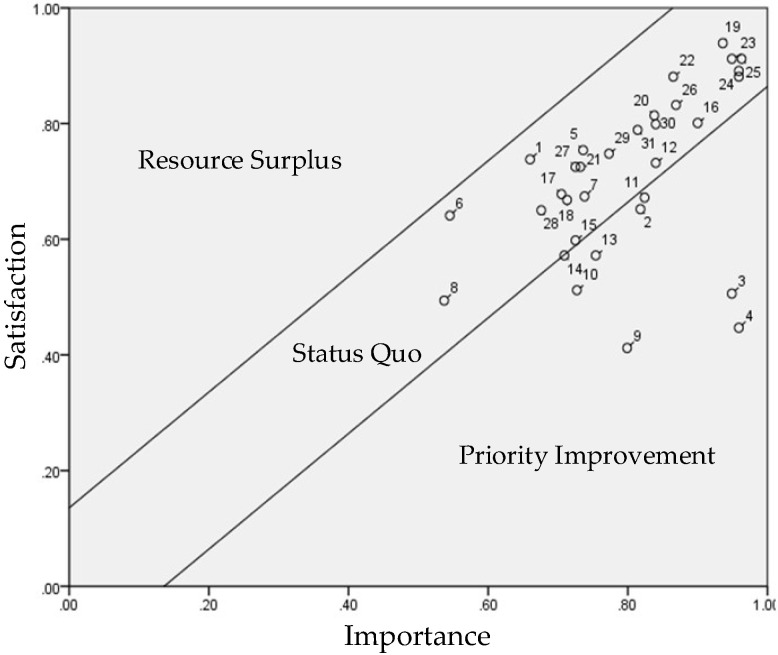
Performance Evaluation Matrix (PEM) of the Female Respondents.

We incorporated the importance and satisfaction values of the male and female respondents presented in Table 5 into Equations (1) and (2) to standardize the variables and include them in the PEM. We then defined the control lines (Table 6) to illustrate Figure 5. The items in need of improvement from the perspective of the male and female respondents were identified using Figure 5 and Figure 6.

We used Equations (3) and (4) to calculate the population mean and error values of the 32 importance–satisfaction items. In the matrix composed of importance and satisfaction indicators, the population means and error values for the male respondents were 4.009 and 0.136, respectively, and those for the female respondents were 4.009 and 0135, respectively. These values were then incorporated into Equations (5) and (6) to calculate one times the standard deviation of the UCL (0.136) and LCL (−0.136) for the male respondents, as well as the one times the standard deviation of the UCL (0.135) and LCL (−0.135) for the female respondents, as presented in Table 6.

We then incorporated the values in Table 6 into the PEM to identify the items in need of improvement as perceived by the male and female respondents. The items in the priority improvement zone for the male respondents were accessibility (2), learnability (3), usability (4), affordability (9), positioning (10), pedometer (11), sleep tracker (12), and data feedback (14) (Figure 5).

The items in the priority improvement zone for the female respondents were accessibility (2), learnability (3), usability (4), affordability (9), positioning (10), pedometer (11), and heart rate monitor (13) (Figure 6). The results also indicate that the eight abnormal nodes outside the control lines were all related to product attributes. A comparison between the male and female respondents suggests that the female respondents were more concerned with heart rate monitoring, while the male respondents prioritized the improvement of the sleep tracking function.

### 4.5. PEM Analysis for Age

**Figure 7:**
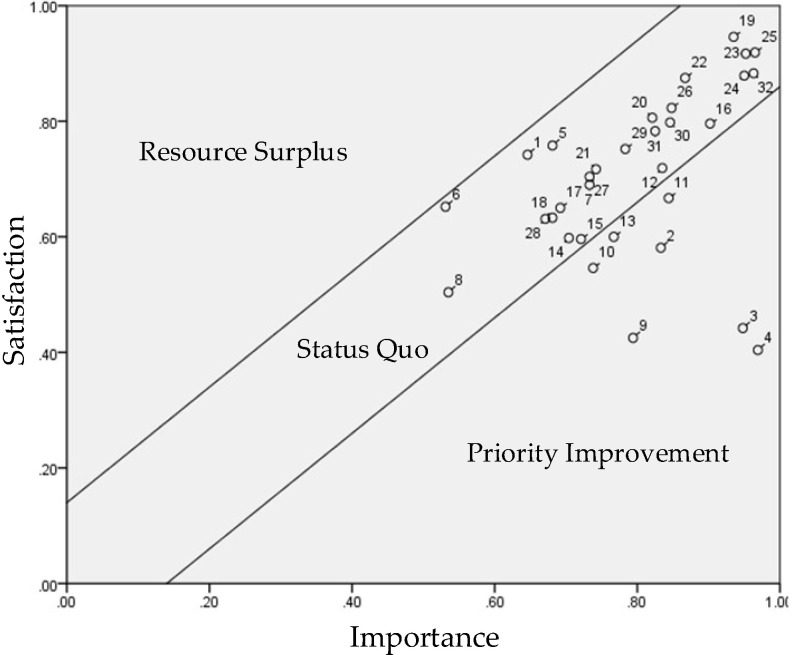
Performance Evaluation Matrix (PEM) of Respondents Younger than 70.

**Figure 8:**
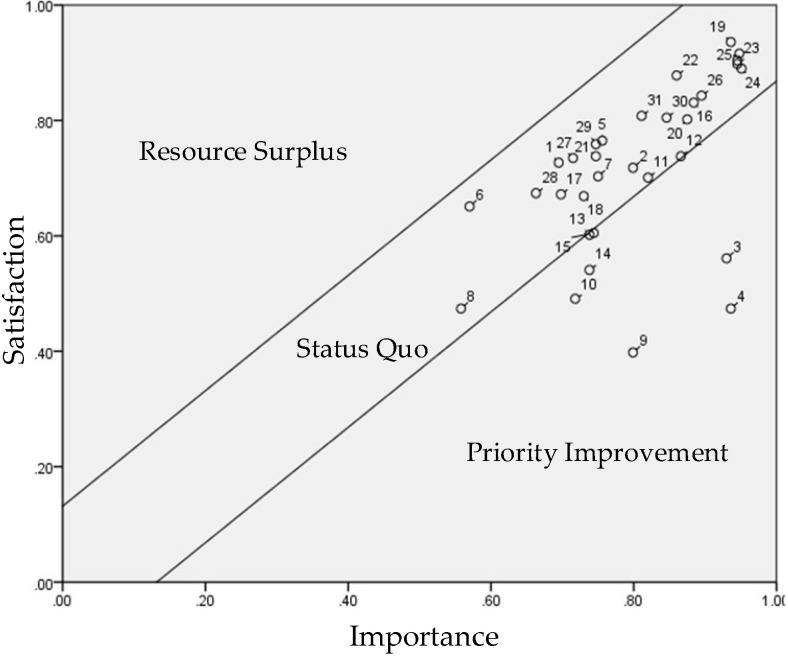
Performance Evaluation Matrix (PEM) of Respondents 70 and Older.

We incorporated the importance and satisfaction values of respondents below and over the age of 70 as presented in Table 7 into Equations (1) and (2) to standardize the variables and include them in the PEM. We then defined the control lines (Table 8) to illustrate Figure 7. The items in need of improvement from the perspective of the male and female respondents were identified using Figure 7 and Figure 8.

We used Equations (3) and (4) to calculate the population mean and error values of the 32 importance–satisfaction items. In the matrix composed of importance and satisfaction indicators, the mean and error values for the respondents younger than 70 were 3.993 and 0.140, respectively, and those for the respondents 70 and older were 4.033 and 0.132, respectively. These values were then incorporated into Equations (5) and (6) to calculate one times the standard deviation of the UCL (0.140) and LCL (−0.140) for the respondents younger than 70, and the one times the standard deviation of the UCL (0.132) and LCL (−0.132) for the respondents 70 and older, as presented in Table 8.

Then, we incorporated the values in Table 8 into the PEM to identify the items in need of improvement as perceived by the respondents younger than 70 and those 70 and older. The items in the priority improvement zone for the respondents younger than 70 were accessibility (2), learnability (3), usability (4), affordability (9), positioning (10), pedometer (11), and heart rate monitor (13) (Figure 7).

The items in the priority improvement zone for the respondents 70 and older were accessibility (2), learnability (3), affordability (9), positioning (10), and data feedback (14). The results indicate that both groups of respondents prioritized the improvement of accessibility and learnability.

### 4.6. Discussion

The main purpose of this study was to determine whether the use of eHealth wearables (smart bracelets) increased older adults’ willingness to engage in casual exercise (regular and fast walking) and improved their life satisfaction. According to the items plotted in the priority improvement zone (high importance zone) of the overall PEM (Figure 3), respondents prioritized eight product attributes of smart bracelets for improvement: accessibility, learnability, usability, affordability, positioning, pedometer, heart rate monitor, and data feedback. Of these, accessibility, learnability, and usability were priority items for improvement in the overall PEM and the demographic PEMs. These were also the product attributes with the lowest satisfaction scores, suggesting that the respondents were concerned about using technological products. This is in line with the finding of Jorunn et al. [34] that product accessibility, learnability, and feedback are key elements influencing older adults’ acceptance of mobile medicine-related technological products.

It is known that older adults are less accepting of technology than young adults since the former find it more difficult to learn and use technology, leading to technology anxiety [12] and rejection. According to the gender PEMs (Figure 5 and Figure 6), the only difference between the male and female respondents’ perception of product attributes was that the male respondents prioritized the improvement of the sleep tracker, while the female respondents prioritized the improvement of the heart rate monitor. The lack of sleep severely affects the quality of life, raises the risk of physical and mental illness, and increases the likelihood of accidents. Besides maintaining good eating habits and minimizing the exposure to risk factors, such as smoking and obesity, wearing eHealth wearables (smart bracelets) to encourage casual exercise (regular or fast walking) is also a feasible approach for maintaining health. A number of previous studies reported the positive impact of smart wearables on disease management and prevention, including diabetes [15] and hypertension [12], and validated the preventive benefits of smart wearables in clinical practice. For example, Saskia et al. [35] found that wearing eHealth wearables improved the life satisfaction of older adults over the age of 55 effectively.

Based on the seven product attributes (i.e., alerts and notifications, security, waterproof and anti-splash, comfort, design, sleep tracker, and calories burned), nine beneficial consequences (i.e., providing respondents with firsthand experiences, the opportunity to learn about smart products and different technical applications, becoming more health-conscious, becoming closer to their families, making more friends, being more relaxed, relieving stress, and satisfying their curiosity), and six personal values (i.e., health improvement, enhanced ability to enjoy life, improved quality of life, improved interpersonal relationships, gaining a life purpose and a sense of social belonging) plotted in the status quo zone (high importance, high satisfaction) of the overall PEM (Figure 3), it is evident that the respondents believed that eHealth wearables are important, and were satisfied with the services provided by such wearables. These items were the factors contributing to product competitiveness. The importance–satisfaction correlation matrix (Table 3) indicated that these product attributes, beneficial consequences, and personal values were significantly and positively correlated, suggesting that eHealth wearables positively influenced older adults’ personal values. These results differ from those of Jeng et al. [24], who developed a means-end chain to examine older adults’ perceived value of mobile health devices and found that product attributes influence beneficial consequences, which in turn influence value targets.

## 5. Conclusions and Suggestions

Faced with the challenges of an aging population, preserving the independence and health of older adults has become an increasingly pressing issue for governments. It has therefore become necessary to pay more attention to the factors contributing to the functional decline of older adults and promote successful aging. eHealth wearables help such adults to monitor their symptoms, strengthen self-health management, and improve their overall health and life satisfaction. However, some of them may underestimate their ability to learn and use the wearables and thus be prejudiced against the use of technological products. Assessing and improving product attributes are therefore required to help them overcome such prejudice and be more accepting of the technology. The findings of this study highlight several feasible promotional strategies for relevant companies.

First, persons with technology anxiety (e.g., older adults) may be the ideal target of these strategies, and companies can market the ease-of-learning and ease-of-use of their products to such potential customers by disclosing detailed market information. Second, companies can draw attention to the accessibility and affordability of their products to create a sense of value-for-money. Third, they can emphasize the beneficial consequences of their products, such as the fact that using them can strengthen family relations or promote relaxation, or stress their health benefits and entertainment value, thereby breaking the stereotype that smart bracelets are cold tech products. Implementation of these strategies will ensure that companies stay abreast of the demands of the older adult market and tweak their products to cater to these demands while at the same time helping such adults to adapt to the technological environment through thoughtful design.

## Figures and Tables

**Table 1:** Questionnaire Dimensions and Items.

Dimensions	Elements	Source
Product attributes	1. Alerts and notifications	Jeng et al. [19] Kotler [29]
	2. Accessibility	
	3. Learnability	
	4. Usability	
	5. Security	
	6. Waterproof and anti-splash	
	7. Comfort	
	8. Design	
	9. Affordability	
	10. Positioning	
	11. Pedometer	
	12. Sleep tracker	
	13. Heart rate monitor	
	14. Data feedback	
	15. Calories burned	
Beneficial consequences	16. Using a smart bracelet, I’ve gained firsthand experiences.	Jeng et al. [19] Gutman [30]
	17. Using a smart bracelet, I’ve learned about smart products.	
	18. Using a smart bracelet, I’ve learned different technical applications.	
	19. Using a smart bracelet, I’m more health-conscious.	
	20. Using a smart bracelet, I’ve become closer to my family.	
	21. Using a smart bracelet, I’ve made more friends.	
	22. Using a smart bracelet, I’m more relaxed.	
	23. Using a smart bracelet, I’m more able to relieve stress.	
	24. Using a smart bracelet has satisfied my curiosity.	
Personal values	25. Using a smart bracelet has improved my health.	Jeng et al. [19] Miele and Parisi [31]
	26. Using a smart bracelet, I’m more able to enjoy life.	
	27. Using a smart bracelet has improved my quality of life.	
	28. Using a smart bracelet has improved my interpersonal relationships.	
	29. Using a smart bracelet has improved my life.	
	30. Using a smart bracelet has brought purpose to my life.	
	31. Using a smart bracelet, I feel a sense of social belonging.	
	32. Using a smart bracelet, I feel a sense of achievement.	

**Table 2:** Reliability Analysis.

Variable	Mean	Standard Deviation	Cronbach’s α
Product attributes (importance)	4.023	0.766	0.631
Beneficial consequences (importance)	4.367	0.554	0.724
Personal values (importance)	4.303	0.562	0.747
Product attributes (satisfaction)	4.403	0.795	0.689
Beneficial consequences (satisfaction)	4.226	0.629	0.688
Personal values (satisfaction)	4.285	0.696	0.822

**Table 3:** Importance and Satisfaction Value of Elements.

Dimensions	Items	$\mu_{x}$	$\mu_{y}$	*Px*	*Py*
Product attributes	1. Alerts and notifications	3.665	3.942	0.666	0.735
	2. Accessibility	4.277	3.553	0.819	0.638
	3. Learnability	4.762	2.966	0.941	0.492
	4. Usability	4.820	2.733	0.955	0.433
	5. Security	3.850	4.044	0.712	0.761
	6. Waterproof and anti-splash	3.189	3.607	0.547	0.652
	7. Comfort	3.961	3.782	0.740	0.695
	8. Design	3.180	2.966	0.545	0.491
	9. Affordability	4.285	2.655	0.796	0.414
	10. Positioning	3.918	3.092	0.729	0.523
	11. Pedometer	4.335	3.723	0.834	0.681
	12. Sleep tracker	4.193	3.908	0.848	0.727
	13. Heart rate monitor	4.019	3.403	0.755	0.601
	14. Data feedback	3.874	3.296	0.718	0.574
	15. Calories burned	3.922	3.398	0.731	0.600
Beneficial consequences	16. Using a smart bracelet, I’ve gained firsthand experiences.	4.578	4.243	0.894	0.811
	17. Using a smart bracelet, I’ve learned about smart products.	3.777	3.636	0.694	0.659
	18. Using a smart bracelet, I’ve learned different technical applications.	3.806	3.592	0.701	0.648
	19. Using a smart bracelet, I’m more health-conscious.	4.743	4.767	0.936	0.942
	20. Using a smart bracelet, I’ve become closer to my family.	4.374	4.218	0.843	0.805
	21. Using a smart bracelet, I’ve made more friends.	3.976	3.903	0.744	0.726
	22. Using a smart bracelet, I’m more relaxed.	4.456	4.505	0.864	0.876
	23. Using a smart bracelet, I’m more able to relieve stress.	4.796	4.636	0.949	0.909
	24. Using a smart bracelet has satisfied my curiosity.	4.801	4.534	0.950	0.883
Personal values	25. Using a smart bracelet has improved my health.	4.830	4.670	0.958	0.917
	26. Using a smart bracelet, I’m more able to enjoy life.	4.471	4.325	0.868	0.831
	27. Using a smart bracelet has improved my quality of life.	3.903	3.869	0.726	0.717
	28. Using a smart bracelet has improved my interpersonal relationships.	3.670	3.597	0.667	0.649
	29. Using a smart bracelet has improved my life.	4.073	4.019	0.768	0.755
	30. Using a smart bracelet has brought purpose to my life.	4.384	4.204	0.846	0.801
	31. Using a smart bracelet, I feel a sense of social belonging.	4.277	4.175	0.819	0.794
	32. Using a smart bracelet, I feel a sense of achievement.	4.820	4.568	0.955	0.892

*μx*: the mean value of importance; *μy*: the mean value of satisfaction; *Px*: the percentage index value for importance; *Py*: the percentage index value for satisfaction_._

**Table 4:** Index Values of the Overall Performance Evaluation Matrix PEM.

	Index Value	Population Mean (*µ*)	Population Error (*σ*)	UCL (1 × *σ*)	LCL (1 × *σ*)
Performance Evaluation					
Importance vs. Satisfaction		4.010	0.136	+0.136	−0.136

UCL: Upper Control Line; LCL: Lower Control Line.

**Table 5:** Importance and Satisfaction Value of Elements Based on Gender.

Dimension	Elements	$\mu_{x}$		$\mu_{y}$		*Px*		*Py*	
		Male	Female	Male	Female	Male	Female	Male	Female
Product attributes	1. Alerts and notifications	3.702	3.639	3.929	3.951	0.676	0.660	0.732	0.738
	2. Accessibility	4.286	4.271	3.476	3.607	0.821	0.818	0.619	0.652
	3. Learnability	4.714	4.795	2.881	3.025	0.929	0.949	0.470	0.506
	4. Usability	4.798	4.836	2.655	2.787	0.949	0.959	0.414	0.447
	5. Security	3.714	3.943	4.083	4.016	0.679	0.736	0.771	0.754
	6. Waterproof and anti-plash	3.202	3.180	3.667	3.566	0.551	0.545	0.667	0.641
	7. Comfort	3.976	3.951	3.905	3.697	0.744	0.738	0.726	0.674
	8. Design	3.226	3.148	2.952	2.975	0.557	0.537	0.488	0.494
	9. Affordability	4.167	4.197	2.667	2.648	0.792	0.799	0.417	0.412
	10. Positioning	3.929	3.910	3.155	3.049	0.732	0.727	0.539	0.512
	11. Pedometer	4.393	4.295	3.774	3.689	0.848	0.824	0.693	0.672
	12. Sleep tracker	4.441	4.361	3.881	3.926	0.860	0.840	0.720	0.732
	13. Heart rate monitor	4.024	4.016	3.571	3.287	0.756	0.754	0.643	0.572
	14. Data feedback	3.929	3.836	3.310	3.287	0.732	0.709	0.577	0.572
	15. Calories burned	3.952	3.902	3.405	3.393	0.738	0.725	0.601	0.598
Beneficial consequences	16. Using a smart bracelet, I’ve gained firsthand experiences.	4.548	4.598	4.298	4.205	0.887	0.900	0.824	0.801
	17. Using a smart bracelet, I’ve learned about smart products.	3.714	3.820	3.524	3.713	0.679	0.705	0.631	0.678
	18. Using a smart bracelet, I’ve learned different technical applications.	3.738	3.853	3.476	3.672	0.685	0.713	0.619	0.668
	19. Using a smart bracelet, I’m more health-conscious.	4.738	4.746	4.786	4.754	0.935	0.936	0.946	0.939
	20. Using a smart bracelet, I’ve become closer to my family.	4.405	4.353	4.167	4.254	0.851	0.838	0.792	0.814
	21. Using a smart bracelet, I’ve made more friends.	4.048	3.926	3.905	3.902	0.762	0.732	0.726	0.725
	22. Using a smart bracelet, I’m more relaxed.	4.452	4.459	4.476	4.525	0.863	0.865	0.869	0.881
	23. Using a smart bracelet, I’m more able to relieve stress.	4.798	4.795	4.619	4.648	0.949	0.949	0.905	0.912
	24. Using a smart bracelet has satisfied my curiosity.	4.750	4.836	4.488	4.566	0.938	0.959	0.872	0.891
Personal values	25. Using a smart bracelet has improved my health.	4.798	4.853	4.702	4.648	0.949	0.963	0.926	0.912
	26. Using a smart bracelet, I’m more able to enjoy life.	4.464	4.475	4.321	4.328	0.866	0.869	0.830	0.832
	27. Using a smart bracelet has improved my quality of life.	3.905	3.902	3.821	3.902	0.726	0.725	0.705	0.725
	28. Using a smart bracelet has improved my interpersonal relationships.	3.619	3.705	3.595	3.598	0.655	0.676	0.649	0.650
	29. Using a smart bracelet has improved my life.	4.048	4.090	4.060	3.992	0.762	0.773	0.765	0.748
	30. Using a smart bracelet has brought purpose to my life.	4.417	4.361	4.214	4.197	0.854	0.840	0.804	0.799
	31. Using a smart bracelet, I feel a sense of social belonging.	4.310	4.254	4.202	4.156	0.827	0.814	0.801	0.789
	32. Using a smart bracelet, I feel a sense of achievement.	4.798	4.836	4.631	4.525	0.949	0.959	0.908	0.881

*μx*: the mean value of importance; *μy*: the mean value of satisfaction; *Px*: the percentage index value for importance; *Py*: the percentage index value for satisfaction_._

**Table 6:** Index Values for Gender in the Performance Evaluation Matrix (PEM).

	Index Value	Population Mean (*µ*)		Population Error (*σ*)		UCL (1 × *σ*)		LCL (1 × *σ*)	
Performance Evaluation		Male	Female	Male	Female	Male	Female	Male	Female
Importance vs. satisfaction		4.009	4.009	0.136	0.135	+0.136	+0.135	−0.136	−0.135

UCL: Upper Control Line; LCL: Lower Control Line.

**Table 7:** Performance Values of the Importance–Satisfaction Variables Based on Age.

Construct	Item	$\mu_{x}$		$\mu_{y}$		*Px*		*Py*	
		Younger than 70	70 and Older	Younger than 70	70 and Older	Younger than 70	70 and Older	Younger than 70	70 and Older
Product attributes	1. Alerts and notifications	3.583	3.779	3.967	3.907	0.646	0.695	0.742	0.727
	2. Accessibility	4.333	4.198	3.325	3.872	0.833	0.799	0.581	0.718
	3. Learnability	4.792	4.721	2.767	3.244	0.948	0.930	0.442	0.561
	4. Usability	4.875	4.744	2.617	2.895	0.969	0.936	0.404	0.474
	5. Security	3.725	4.023	4.033	4.058	0.681	0.756	0.758	0.765
	6. Waterproof and anti-splash	3.125	3.279	3.608	3.605	0.531	0.570	0.652	0.651
	7. Comfort	3.933	4.000	3.758	3.814	0.733	0.750	0.690	0.703
	8. Design	3.142	3.233	3.017	2.895	0.535	0.558	0.504	0.474
	9. Affordability	4.175	4.198	2.700	2.593	0.794	0.799	0.425	0.398
	10. Positioning	3.950	3.872	3.183	2.965	0.738	0.718	0.546	0.491
	11. Pedometer	4.375	4.279	3.667	3.802	0.844	0.820	0.667	0.701
	12. Sleep tracker	4.342	4.465	3.875	3.954	0.835	0.866	0.719	0.738
	13. Heart rate monitor	4.067	3.954	3.400	3.407	0.767	0.738	0.600	0.602
	14. Data feedback	3.817	3.954	3.392	3.163	0.704	0.738	0.598	0.541
	15. Calories burned	3.883	3.977	3.383	3.419	0.721	0.744	0.596	0.605
Beneficial consequences	16. Using a smart bracelet, I’ve gained firsthand experiences.	4.608	4.535	4.183	4.326	0.902	0.884	0.796	0.831
	17. Using a smart bracelet, I’ve learned about smart products.	3.767	3.791	3.600	3.686	0.692	0.698	0.650	0.672
	18. Using a smart bracelet, I’ve learned different technical applications.	3.725	3.919	3.533	3.674	0.681	0.730	0.633	0.669
	19. Using a smart bracelet, I’m more health-conscious.	4.742	4.744	4.783	4.744	0.935	0.936	0.946	0.936
	20. Using a smart bracelet, I’ve become closer to my family.	4.283	4.500	4.225	4.209	0.821	0.875	0.806	0.802
	21. Using a smart bracelet, I’ve made more friends.	3.967	3.988	3.867	3.954	0.742	0.747	0.717	0.738
	22. Using a smart bracelet, I’m more relaxed.	4.467	4.442	4.500	4.512	0.867	0.860	0.875	0.878
	23. Using a smart bracelet, I’m more able to relieve stress.	4.808	4.779	4.667	4.593	0.952	0.945	0.917	0.898
	24. Using a smart bracelet has satisfied my curiosity.	4.800	4.802	4.517	4.558	0.950	0.951	0.879	0.890
Personal values	25. Using a smart bracelet has improved my health.	4.858	4.791	4.675	4.663	0.965	0.948	0.919	0.916
	26. Using a smart bracelet, I’m more able to enjoy life.	4.392	4.581	4.292	4.372	0.848	0.895	0.823	0.843
	27. Using a smart bracelet has improved my quality of life.	3.933	3.861	3.817	3.942	0.733	0.715	0.704	0.735
	28. Using a smart bracelet has improved my interpersonal relationships.	3.683	3.651	3.525	3.698	0.671	0.663	0.631	0.674
	29. Using a smart bracelet has improved my life.	4.133	3.988	4.008	4.035	0.783	0.747	0.752	0.759
	30. Using a smart bracelet has brought purpose to my life.	4.383	4.384	4.192	4.221	0.846	0.846	0.798	0.805
	31. Using a smart bracelet, I feel a sense of social belonging.	4.300	4.244	4.133	4.233	0.825	0.811	0.783	0.808
	32. Using a smart bracelet, I feel a sense of achievement.	4.850	4.779	4.533	4.616	0.963	0.945	0.883	0.904

*μx*: the mean value of importance; *μy*: the mean value of satisfaction; *Px*: the percentage index value for importance; *Py*: the percentage index value for satisfaction_._

**Table 8:** Index Values for Age in the Performance Evaluation Matrix (PEM).

	Index	Population Mean (*µ*)		Population Error (*σ*)		UCL (1 × *σ*)		LCL (1 × *σ*)	
Evaluation		Younger than 70	70 and Older	Younger than 70	70 and Older	Younger than 70	70 and Older	Younger than 70	70 and Older
Importance vs. Satisfaction		3.993	4.033	0.140	0.132	+0.140	+0.132	−0.140	−0.132

UCL: Upper Control Line; LCL: Lower Control Line.
